# Using geographic information system to evaluate land use and land cover affected by flooding in Adamawa State, Nigeria

**DOI:** 10.4102/jamba.v11i1.494

**Published:** 2019-01-10

**Authors:** Salihu D. Musa, Terwase Shabu

**Affiliations:** 1 Department of Geography and Planning, Kogi State University, Nigeria; 2 Department of Geography, Benue State University, Nigeria

## Abstract

The impact of natural disaster on land use and/or land cover (LULC) has emerged as a global phenomenon and is perhaps the most significant regional anthropogenic and natural disturbance to the environment. Natural disasters and land use change are major concerns all over the world, and if these two concerns exist together along large rivers, then the consequences for people and the human activities may be severe. This study evaluated land use and land cover affected by flooding along Benue River in Adamawa State, Nigeria. Satellite imageries of land use, vegetation, settlements and drainage basins were collected from the National Space Research and Development Agency, Abuja, and the National Centre for Remote Sensing, Jos. The imageries were collected for the selected study period: 2002, 2012 and 2014, all between October and November. The three imageries were used to generate a LULC map of the study areas. Land cover and/or land use and flood inundation analysis was carried out using Integrated Land and Water Information System image processing and Earth Resources Data Analysis (ERDAS 9.2) image processing geographic information system software. A supervised classification was done using the LULC samples collected on the field. This final product generated a LULC map for the entire study area. Six LULC classes were generated: waterbodies, farmland, vegetation, settlement, alluvial deposits and bareland. The study revealed that in 2002 floods affected 38% farmland and 53% vegetation of the total inundated areas. In 2012, it affected 56% farmland cover and 35% vegetation, while in 2014, it affected 51% farmland cover and 42% vegetation cover of the total inundated area of Adamawa State. This study concluded that the most flood-affected land cover is farmland and recommended that farming activities should be located outside flood-prone areas in order to avoid food insecurity issues during and after flood disaster in the state.

## Introduction

Flood strikes communities around the globe each year. It is one of the top ten natural disasters in the world in terms of people affected and property damage caused (Miller 2005). Flooding is a major problem and a risk to environmental components of the society. In humid tropical climates, river flooding is a recurrent and natural phenomenon (Dewan 2013; PDNA 2013; Sanyal& Lu 2004), causing damage to social and economic activities, agricultural land and crops. This limits the capacity of livelihoods to recover and reconstruct after flood events.

Ologunorisa (2004) defined a flood as a covering by water on land not normally covered by water. According to Oyegbile (2008), flood refers to an overflow of water that submerges land. This is not entirely correct as rivers and the sea also experience floods. Flooding is the unusual presence of water in a place to an intensity that has a disruptive effect on normal activities. Flooding arises mainly as a result of overflowing rivers and heavy rain over a short duration. In the sense of ‘flowing water’, flooding may also be applied to the inflow of tide on land and may also result from the excessive volume of water within a body of water, such as a river or lake, which flows or breaks levees, with the result that some of the water escapes its boundaries. It may also be because of accumulation of rainwater on saturated ground in an area (Oyegbile 2008). Natural disasters are defined as (UNISDR 2004):

a serious disruption of the functioning of society, causing widespread human, material or environmental losses which exceed the capacity of the affected society to cope using only its own resources. (p. 5)

The areas along the bank of the Benue River and its tributaries are flood disaster prone because of their geographical location, that is, land characteristics.

Land use has been seen as a product of interactions between a society’s cultural background, skills and physical needs on the one hand and the natural potential of land on the other hand (Nagamani & Ramachandran 2003). According to the Food and Agriculture Organization of the United Nations, ‘Land use is characterized by the arrangements, activities and inputs people undertake in a certain land cover type’ (FAO 2000). According to this definition, land use reflects human activities such as the use of the land in industrial zones, residential zones and agricultural fields. Land and land use may be affected differently by various types of disaster. Large areas maybe left uninhabitable by long-term inundation through floods, storm surges and tsunamis. Cyclones, tornados and other big wind events do not necessarily have a large physical effect on land but may cause destruction of houses and resources and displacement of a large number of people (UN-HABITAT 2010). Land use and land cover along the bank of the Benue River in Adamawa State of Nigeria affected by flooding are diverse. Land in these areas is used intensively for agriculture, settlements, forests, ponds, waterbodies and fisheries production, industrial and infrastructure developments, and tourism (Islam et al. 2016). Moreover, construction of dams in the upper part of the river channel is considered as one of the main land use activities that has changed the stability of land covers along the banks of the river in terms of water flow activities.

The principal flood problem in the flood plains of the Benue River is damage to agricultural land and crops. During the rainy season, floodwaters overflow the banks of low-capacity channels and inundate thousands of hectares (ha) of adjacent cropland. Rainstorms also produce stream flows above channel capacities that cause sheet-water flooding (Banerjee 2006; Khan, Mia & Hossain 2012; Leitch & Scott 1977). These floods result in serious reductions in agricultural production, which in turn have a depressing effect on the economy of the affected regions (Leitch& Scott 1977).

People settle on floodplains because of the many advantages, including fertile soil for farming, ample water for irrigation, availability of nearby rivers for transportation and recreation, flat land suitable for crop production and other economic activities (Miller 2005). Floodplains provide the world’s most productive farmland as a result of nutrient-rich silt left after flood water recedes, but these areas are bedevilled with natural disasters. Floodplains within the Benue trough in Nigeria are characterised with flood events (Etuonovbe 2011). Heavy rainfall combined with increased runoff has increased the severity of flooding along the Benue River and its tributaries within Adamawa State (PDNA 2013). Apart from precipitation, several other factors influence the generation of surface runoff within the region, which eventually leads to flooding. Among the most important of these factors are geology, land use, topography, soil characteristics, vegetation and evapotranspiration (Duru & Chibo 2014). Even with this impending danger of flood disaster, many within the region have little choice but try to survive in flood-prone areas.

In contemporary time, flooding has become a common characteristic and part of life in Nigeria not only in the low-lying coastal areas such as Lagos, Port Harcourt, Calabar and Warri, but also in the hinterland (Oriola 2000). Upland places such as Kano, Sokoto, Maiduguri and Bauchi often experience splash floods during heavy rainfall events. The rural areas are also not left out from this environmental danger.

The incident of floods is becoming a reoccurring phenomenon in most areas, leading to huge loss of properties and lives. For example, in 1973, 1974 and 1976, cases of floods were recorded in Ilorin (Jimoh 2000); in 1973, 1980 and 2011 Ogunpa floods in Ibadan occurred (Amori et al. 2012). In Adamawa State, flood has almost become an annual event, leading to loss and destruction of properties worth millions of Naira. In 1996, 2000, 2002, 2005, 2007, 2008, 2012 and 2014, floods occurred in different parts of the state, especially in areas along the Benue River.

A disastrous flood in September 2012 affected about 27 states of the federation, covered one-third of arable land for months, killed more than 300 people and displaced about 2 million people from their homes (PDNA 2013). It affected farmland used for crop production, mainly vegetable gardening and other crops such as maize, sorghum, millet, groundnut, beans, yam, cassava and sweet potatoes along the banks of the Benue River and its distributaries. The communities within the region have a limited capacity to control the hydrological events ensuing from the basin catchment areas, thereby increasing their vulnerability to flood disaster.

Agriculture is considered the engine of the community’s economy in Adamawa State. It is worth noting that the agricultural sector is the highest employer of the affected household labour force (Gonslaves & Mohan 2011). Flood impact on farming activities and commercial value of agriculture can certainly culminate in a serious agrarian change that would impact the greater number of people and the livelihood strategy of farming communities in the area.

Previous studies on flooding within the area focused on causes, health effects of incidence, government control and resident coping measures (Mngutyo & Ogwuche 2013; Ocheri & Okele 2012; Ologunorisa & Terso 2006; Shabu & Tyonum 2013). Some of the studies looked at the social and economic impact of flooding (Duru & Chibo 2014; Oruonye 2012; Tadesse 2009). Elsewhere, studies on natural disaster and land use and/or land cover (LULC) were carried out to ascertain the impact of natural disaster, especially flooding, on LULC (Khan 2012; Krishnakumar et al. 2011; UN-HABITAT 2010). However, there is no research that has covered flood impact on LULC in Adamawa State of the upper Benue trough as a hydrological area.

## Methodology

Satellite imageries of land use, vegetation, settlements and drainage basins were collected from the National Space Research and Development Agency and the National Centre for Remote Sensing, Jos. Land cover and flood inundation analysis was carried out using Integrated Land and Water Information System (ILWIS) image processing and Earth Resources Data Analysis (ERDAS 9.2) image processing geographic information system (GIS) software. The process and procedure involved in the application of ILWIS and ERDAS image processing software are presented next.

The imageries were collected for the selected study period, namely, 2002, 2012 and 2014, all between October and November. A few of the downloaded images have cloud cover distortions, while most of them are cloud free. The Landsat imageries downloaded from the Global Land Cover Facility (GLCF) website are in 11 bands; the appropriate bands (bands 5, 4 and 3) were selected stacked as ‘green’, ‘red’ and ‘blue’ bands. This was stacked to form one false colour composite image. Lastly, all data were projected to World Geographic System (WGS), Northern Hemisphere and Zone 32 projection. This was done in order to have a homogenous projection.

**FIGURE 1:**
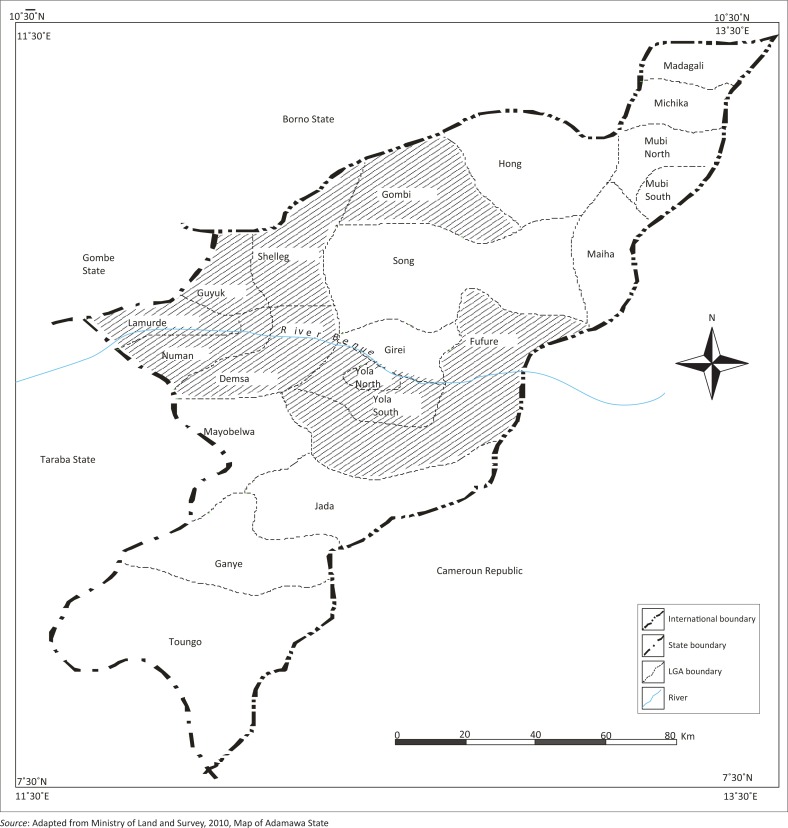
Adamawa State showing the study area.

Two factors were considered in modelling the inundated areas in the study area (see Figure 1): proximity to the Benue River and elevation. The elevation data obtained from the field were used to ascertain the highest flooded point observed in 2012; this data was used to perform a slicing operation in ILWIS using the Shuttle Radar Topography Mission 1 (SRTM1) data to establish the area flooded and non-flooded in 2012. The areas with the highest elevations obtained from the field were the areas that are considered non-flooded, while the elevations below the highest point were considered flooded. The operation was performed for all different sub-basins using terrain height as a major factor for basin delineation.

Furthermore, a domain group in ILWIS was created with a flood level at the highest levels recorded. A flood inundation map was created by using the slicing algorithm in which the *SRTM* Digital Elevation Model (DEM) was sliced by the highest flood level mark in the domain group.

The three imageries were used to generate a LULC map of the study areas. Earth Resources Data Analysis imagine 9.2 software was used for this analysis. The imageries were classified using maximum likelihood classification method. A supervised classification was done using the LULC samples collected on the field. This final product generated an LULC map for the entire study area. Six LULC classes were generated: water bodies, farmland, vegetation, settlement, alluvial deposits and bareland.

The flood inundation map was used to mask out LULCs that were affected. This automatically generates a layer depicting LULC elements affected by flood disasters along the Benue River in Adamawa State. The total area of LULC in the study areas for 2002, 2012 and 2014 was estimated in *Excel* and graphs and tables were generated. The area of each class extracted was converted from square meters to hectares. Similar operation was performed for the elements at risk and bar charts were generated to illustrate graphically the LULC areas affected in the 2012 flood disaster.

## Results

From field observation, the entering point of the flooded dam in Gurin Fufora Local Government Area (LGA) of Adamawa State, where the flood height (flood water level) was 197 m. At this point, distance of the flooded water extended to 50 km away from the Benue River. The distance of the flooded water receded to only 8 km in Yola because of narrow flood plains. Beyond Yola at a major tributary, connecting the Benue River, the area of the flooded water increased to about 19 km away from the Benue River. At Numan, because of the contribution from Kirri Dam, the flood water area was about 16 km away from the Benue River. However, where the water exited at Adamawa State the flood water was only 8 km away from the Benue River.

**FIGURE 2:**
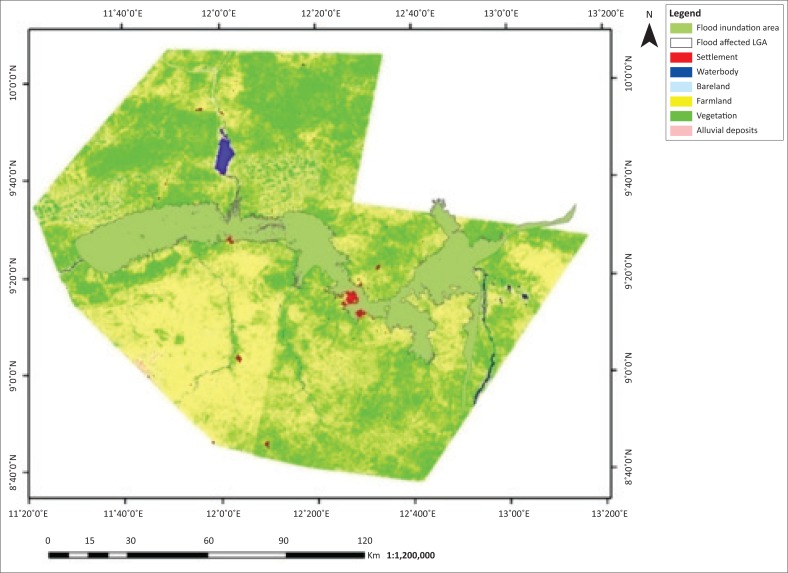
Flood inundation and land use and/or land cover of Adamawa 2002.

**FIGURE 3:**
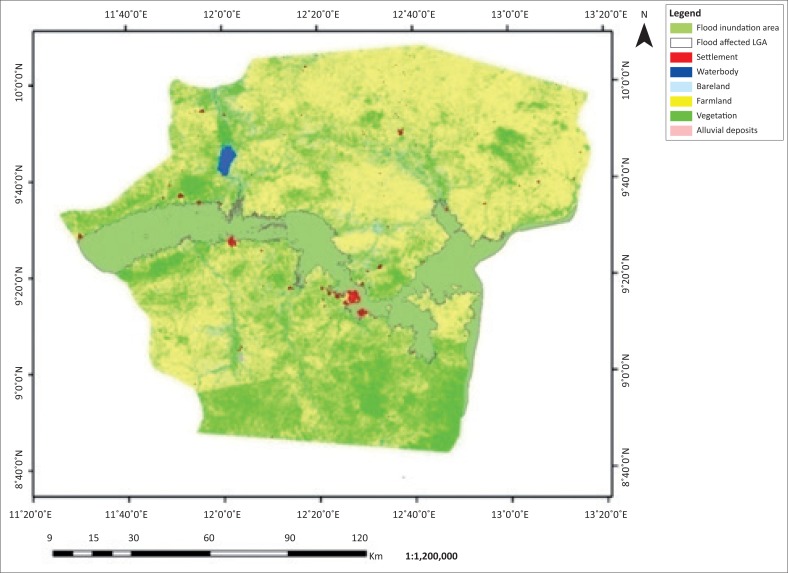
Flood inundation and land use and/or land cover map of Adamawa 2012.

**FIGURE 4:**
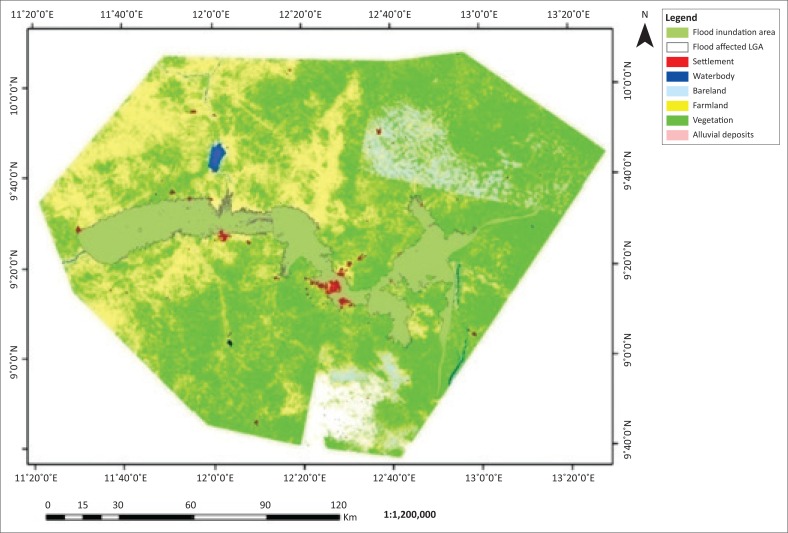
Flood inundation and land use and/or land cover map of Adamawa 2014.

The flooded LGAs in the Adamawa area includes Demsa, Fufore, Girei, Lamurde, Numan, Shelleng, Song, Yola north and Yola south (Figure 1). The flooded area in Adamawa State is about 276 089 ha. Six features, (i.e. waterbody, bareland, farmland, vegetation, alluvial deposits and settlement) of land covers of the affected LGAs, were classified based on their spectral signature. The 2002, 2012 and 2014 classified images are presented in Figures 2–4.

### Land use and/or land cover elements affected by flooding in Adamawa State

**FIGURE 5:**
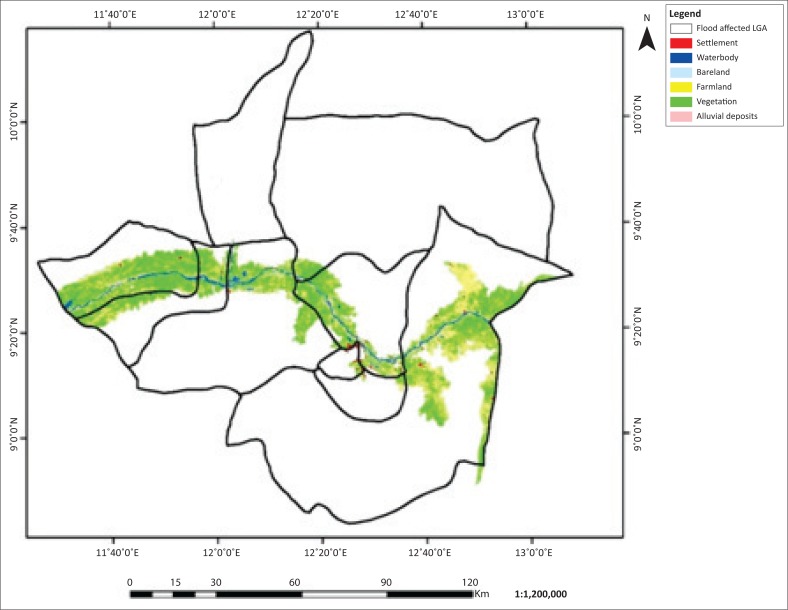
Flooded land cover and/or land use in Adamawa State in 2002.

**FIGURE 6:**
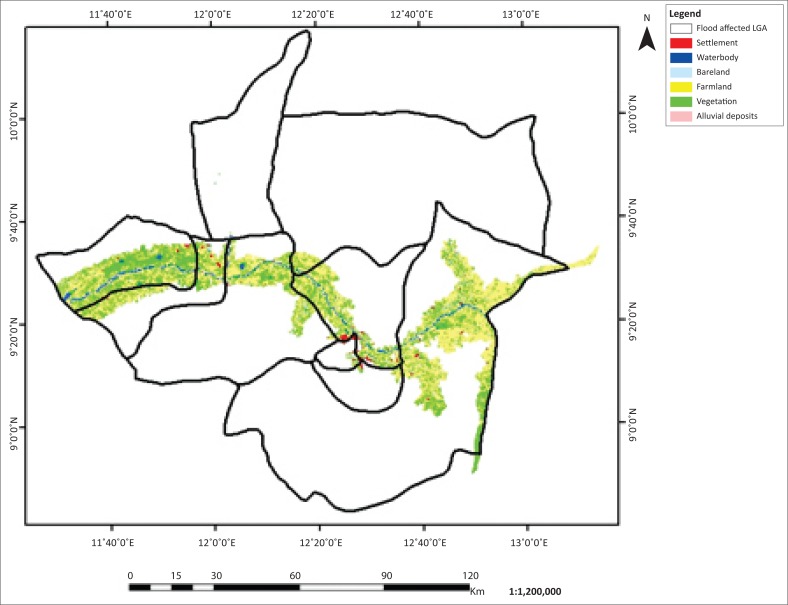
2012 flooded land cover and/or land use in Adamawa State.

**FIGURE 7:**
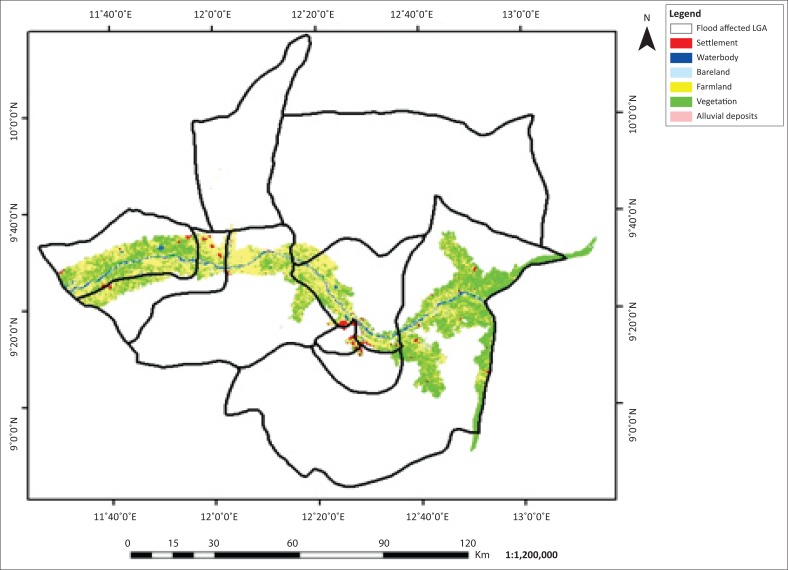
2014 flooded Local Government Area in Adamawa State map.

The classified images of Adamawa covering flood inundated areas of Landsat ETM 2002, 2012 and 2014 are presented in Figures 3, 4 and 5, respectively. Six features of land cover affected by flood waters were identified and estimated. The land covers affected include waterbody, bareland, farmland, vegetation, alluvial deposits and settlement (see Table 1 and Figures 5–7).

**TABLE 1:** Classified area of elements affected by flooding in Adamawa.

Class name	2002 Area (ha)	2012 Area (ha)	2014 Area (ha)
Settlement	0.304371	0.414882	0.570726
Waterbody	1.497180	1.068530	1.0428000
Bareland	0.204426	0.702270	0.114768
Farmland	10.344000	15.551600	14.068500
Vegetation	14.422800	9.641740	11.663700
Alluvial deposits	0.203373	0.208431	0.147960

ha, hectare.

**FIGURE 8:**
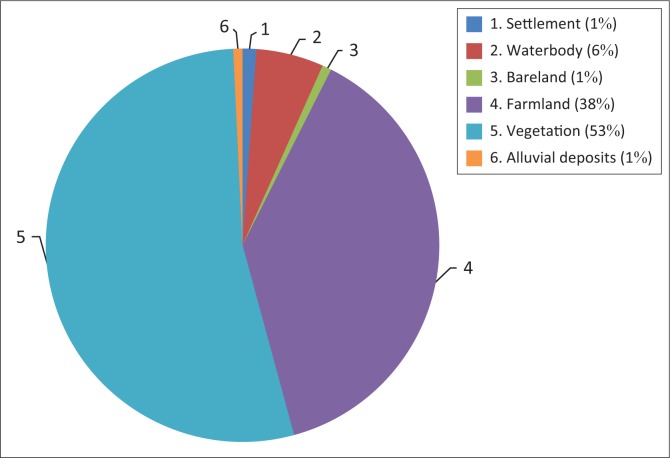
Land cover and land use affected by 2002 flooding in Adamawa.

The flood incidence of 2002 affected 53% vegetation land cover of the area followed by 38% farmland of the total inundated areas of Adamawa State. Only 1% of settlement area was affected by flooding, whereas 6% of waterbody, especially drinking water, was affected by flood disaster (see Figure 8). Apart from 53% of vegetation cover that was affected, 38% of farmland comparing different kinds of crops, such as cereals, roots or tubers, tree crops and others, were affected by flooding of 2002. This led to some extent to food security issues within the areas affected by the flood disaster.

The inundated areas in Adamawa State affected farmland, waterbody, vegetation and alluvial deposits. Among the land cover affected by the 2012 flooding, farmland and vegetative cover were the most affected in the area.

**FIGURE 9:**
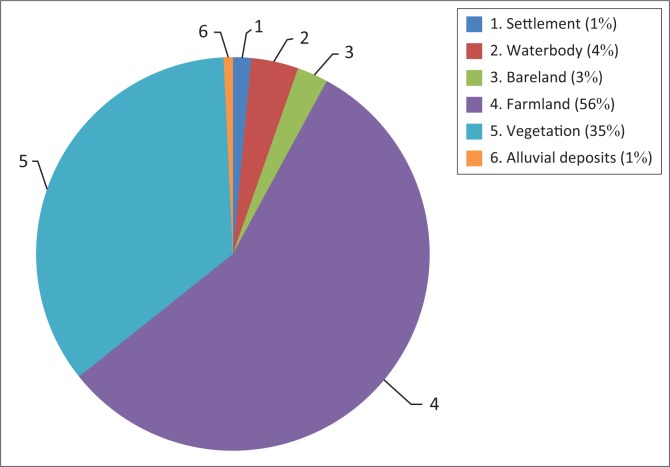
Land cover and/or land use affected by 2012 flooding in Adamawa.

The flood incidence of 2012 affected 56% farmland cover of the area followed by 35% vegetation of the total inundated areas of Adamawa State. Only 2% of settlement area was affected by flooding (see Figure 9). The 2012 flooding affected more farming activities and crop production than any other economic activities. This resulted in food crises and food security issues, leading to malnutrition and diseases in the area.

**FIGURE 10:**
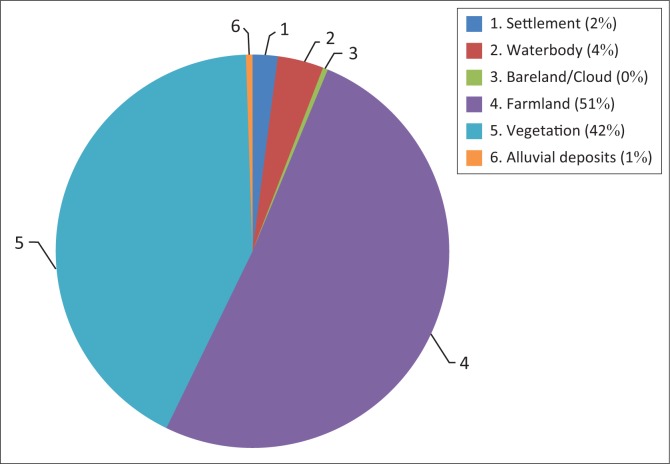
Land cover and/or land use affected by 2002 flooding in Adamawa.

The flood incidence of 2014 affected 51% farmland cover of the area followed by 42% vegetation of the total inundated areas of Adamawa State. Only 2% of settlement area was affected by flooding (see Figure 10). From estimates of land covers affected by the 2014 flooding, it was observed that 51% farmland was affected. Crops at various stages of development were submerged. Even though some of the crops regenerated after flood water resided, their output was not enough to offset losses observed during and after the flood.

### Damage and/or losses to crop production

Crop production is one of the major components usually affected by flood disaster. This is because agricultural activities, especially acculturation of crops, are carried out along the river banks. Soil fertility along this river banks attract the cultivation of different crops by farmers in the region. Therefore, any rise in the volume of water will mean that areas cultivated along the river bank will submerge before other agricultural activities are affected apart from fishing. Out of the sampled affected households, 97.9% and 78.7% in Adamawa and Benue states, respectively, experienced damage and losses in crop production. Information on the farm size of affected crops in the study area is presented in Table 2.

**TABLE 2:** Farm size of affected tree crops.

S/N	Farm size (tree stand)	Adamawa	
		Frequency	%
1	1–50	89	23.3
2	51–100	110	28.8
3	101–150	83	21.7
4	101–200	38	9.9
5	201–250	62	16.2
**Total**	**-**	**382**	**100**

Table 2 shows that in Adamawa State of the upper Benue trough, 28.8% of the affected households experienced damage and/or losses of between 51 and 100 tree stands, followed by 23.3% experienced damage of between l and 50 tree stands. In Benue State of the lower Benue trough, 43.7% of the affected households experienced damage and/or losses of between 101 and 150 tree stands. This is an indication that more tree crops were affected in the lower Benue trough compared to the upper Benue trough, which is based on the fact that tree crops are largely produced in the lower Benue region.

Information in Table 3 shows that 39.5% of the affected households indicated that affected farm size for arable crops was between 1.1 ha and 1.5 ha and 28.5% of the households indicated that arable crops affected were mature for harvest with farm size of between 0.6 ha and 1.0 ha. Another 30.6% of the affected households indicated that about 1.6 ha and 2.0 ha of farms for arable crops were affected by the flood event and 23.0% indicated that their affected arable crops were mature for harvest (farm size 0.1 ha and 0.5 ha) before the flood disaster.

**TABLE 3:** Arable crops affected in Adamawa State.

S/N	Farm size (ha)	Arable crops		Crops mature for harvest	
		*F*	%	*F*	%
1	0.1–0.5	61	16	88	23.0
2	0.6–1.0	39	10.2	109	28.5
3	1.1–1.5	151	39.5	58	15.2
4	1.6–2.0	117	30.6	18	4.7
5	2.1–2.5	11	2.9	48	12.6
6	2.6–3.0	14	3.7	61	16.0
**Total**	**-**	**382**	**100.0**	**382**	**100.0**

ha, hectare; *F*, frequency.

Table 4 shows that 30.9% of the affected households in Adamawa lost between ₦1000.00 and ₦50 000.00 during the flood disaster, followed by 28.3% who lost between ₦51 000.00 and ₦100 000.00 in crop production, while 19.1% of the affected households lost between ₦251 000.00 and ₦300 000.00 in the area of crop production.

**TABLE 4:** Estimated losses in crop production.

S/N	Amount (*₦*)	Adamawa	
		Frequency	%
1	1000.00–50 000.00	118	30.9
2	51 000.00–100 000.00	108	28.3
3	101 000.00–150 000.00	59	15.4
4	151 000.00–200 000.00	7	1.8
5	201 000.00–250 000.00	17	4.5
6	251 000.00–300 000.00	73	19.1
**Total**	**-**	**382**	**100.0**

**₦,** Nigerian Naira (currency).

### Number of working days lost in agricultural sector

Employment was also affected by the flood disaster. Work premises that were submerged by flood water or access roads to work premises were affected, restricting both employers and employees from going to work. Information on the number of working days lost during the flooding is presented in Table 5.

**TABLE 5:** Number of working days lost in agricultural sector in Adamawa.

Number of days	Crop production		Fisheries		Livestock production		Agro-processing	
	*F*	%	*F*	%	*F*	%	*F*	%
No loss	19	5.0	19	5.0	17	4.5	33	8.6
1–7 days	10	2.6	4	1.0	6	1.6	9	2.4
8–14 days	3	< 1.0	2	< 1.0	5	1.3	7	1.8
15–21 days	3	< 1.0	4	1.0	52	13.6	3	< 1.0
22–28days	39	10.2	40	10.9	00	0.0	44	11.5
29–35 days	308	80.6	313	81.9	302	79.1	286	74.9
**Total**	**382**	**100.0**	**382**	**100.0**	**382**	**100.0**	**382**	**100.0**

*F*, frequency.

Table 5 shows that 80.6% of the affected households in the upper Benue trough lost between 29 and 35 days in crop production, while 81.9% lost in fisheries, 79.1% lost in livestock production and 74.9% lost in agro-processing for the same number of days.

### Estimated income loss in agricultural sector

The many advantages of flood plains, which include fertile soil, ample water for irrigation and rivers for transportation, attract farming activities to vulnerable areas. These areas are prone to flood disasters which can occur at any time, and farmers as a result experience losses and damages in crops and/or animal production and in the agro-processing sectors. From the survey conducted, it was asserted that farmers experienced some level of income loss in the sector. Information on estimated income loss in the area is presented in Table 6.

**TABLE 6:** Income loss in agricultural sector.

Income (*₦*)	Crop production		Fisheries		Livestock production		Agro-processing	
	*F*	%	*F*	%	*F*	%	*F*	%
100.00–301 000.00	36	9.4	26	6.8	172	45.0	158	41.4
301 000.00–600 000.00	89	23.3	83	21.7	16	4.2	18	4.7
601 000.00–900 000.00	164	42.9	77	20.2	101	26.4	72	18.8
901 000.00–1 200 000.00	37	9.7	117	30.6	28	7.8	48	12.6
No loss	56	14.6	79	20.7	65	17.0	86	22.5

**₦,** Nigerian Naira (currency); *F*, frequency.

Table 6 shows that in Adamawa State of the upper Benue trough, 42.9% of the affected households lost between ₦601 000.00 and ₦900 000.00 and 23.3% lost between ₦301 000.00 and ₦600 000.00 as a result of flooding, while only 14.6% of the affected households did not experience income loss during the flooding in the area of crop production. In fisheries production, 30.6% of the affected households lost between ₦901 000.00 and ₦1 200 000.00, 20.2% lost between ₦901 000.00 and ₦1 200 000.00 because of flooding, while 20.7% did not experience income loss in the area. Also, in animal production, 45.0% of the affected households did not experience income loss in this area of agricultural production. Agro-processing component of the agricultural sector experienced little income loss. Out of the sample affected households, 41.4% lost between ₦1000.00 and ₦300 000.00, 18.3% lost between ₦601 000.00 and ₦900 000.00, while 22.5% did not experience income loss in the area of agro-processing.

## Conclusion

Land along the river banks is usually fertile and suitable for crop production because of the nutrient-rich silt deposited after flood water recedes. However, these areas along the rivers are characterised by flood disaster. Moreover, the flood events return period has declined from 10 years to less than 10 years in recent times as a result of climate change. Farmland cover in these areas is frequently affected by flood disaster and as such the following is required to minimise the adverse effect of the disaster on farming activities in the area: avoidance of farming activities in flood-prone areas in rainy season, avoidance of areas that drain slowly after intense or heavy rainfall, and cultivation of crop species that are tolerant to long period of inundation in flood risk areas. We can advocate for use of indigenous techniques for people living with floods to protect crops and animals. There is a need to build their capacities to anticipate, cope and recover as they will persist to cultivate on flood plains. These measures can minimise food shortage challenges experienced during and after flood disaster in the study area.

Flood inundated areas of 2002, 2012 and 2014 affected majorly farmlands, vegetation cover and settlements. This severely affected the agricultural sector in the state, most especially crop production which is the major livelihood strategy in the area. Flood damage to crop and agricultural land deteriorates societies’ and communities’ ability to recover their livelihoods, security and health status. There is a need for communities to adapt proactive measures to minimise the damage and loss of crops through capacities to anticipate, cope with and recover from such devastating events. This can help communities become versatile in pooling resources through community-based efforts in issues of preparedness and recovery measures and at the same time in minimising damage and losses to the food the production sector to ensure food security in the area.
